# Higher circulating intermediate monocytes are associated with cognitive function in women with HIV

**DOI:** 10.1172/jci.insight.146215

**Published:** 2021-06-08

**Authors:** Rebecca T. Veenhuis, Dionna W. Williams, Erin N. Shirk, Celina M. Abreu, Edna A. Ferreira, Jennifer M. Coughlin, Todd T. Brown, Pauline M. Maki, Kathryn Anastos, Joan W. Berman, Janice E. Clements, Leah H. Rubin

**Affiliations:** 1 Department of Molecular and Comparative Biology,; 2 Division of Clinical Pharmacology,; 3 Department of Psychiatry and Behavioral Sciences, and; 4 Department of Medicine, Johns Hopkins University School of Medicine, Baltimore, Maryland, USA.; 5 Department of Epidemiology, Bloomberg School of Public Health, Johns Hopkins University, Baltimore, Maryland, USA.; 6 Department of Psychiatry, College of Medicine, and Department of Psychology, College of Liberal Arts and Sciences, University of Illinois at Chicago, Chicago, Illinois, USA.; 7 Department of Medicine and Epidemiology & Population Health, and; 8 Department of Pathology and Department of Microbiology & Immunology, Albert Einstein College of Medicine, Bronx, New York, USA.; 9 Department of Pathology and; 10 Department of Neurology, Johns Hopkins University School of Medicine, Baltimore, Maryland, USA.

**Keywords:** AIDS/HIV, Immunology, Monocytes, Neurological disorders

## Abstract

**BACKGROUND:**

Identifying a quantitative biomarker of neuropsychiatric dysfunction in people with HIV (PWH) remains a significant challenge in the neuroHIV field. The strongest evidence to date implicates the role of monocytes in central nervous system (CNS) dysfunction in HIV, yet no study has examined monocyte subsets in blood as a correlate and/or predictor of neuropsychiatric function in virally suppressed PWH.

**METHODS:**

In 2 independent cohorts of virologically suppressed women with HIV (vsWWH; *n* = 25 and *n* = 18), whole blood samples were obtained either in conjunction with neuropsychiatric assessments (neuropsychological [NP] test battery, self-report depression and stress-related symptom questionnaires) or 1 year prior to assessments. Immune cell subsets were assessed by flow cytometry.

**RESULTS:**

A higher proportion of intermediate monocytes (CD14^+^CD16^+^) was associated with lower global NP function when assessing monocytes concurrently and approximately 1 year before (predictive) NP testing. The same pattern was seen for executive function (mental flexibility) and processing speed. Conversely, there were no associations with monocyte subsets and depression or stress-related symptoms. Additionally, we found that a higher proportion of classical monocytes was associated with better cognition.

**CONCLUSION:**

Although it is widely accepted that lentiviral infection of the CNS targets cells of monocyte-macrophage-microglial lineage and is associated with an increase in intermediate monocytes in the blood and monocyte migration into the brain, the percentage of intermediate monocytes in blood of vsWWH has not been associated with neuropsychiatric outcomes. Our findings provide evidence for a new, easily measured, blood-based cognitive biomarker in vsWWH.

**FUNDING:**

R01-MH113512, R01-MH113512-S, P30-AI094189, R01-MH112391, R01-AI127142, R00-DA044838, U01-AI35004, and P30-MH075673

## Graphical abstract

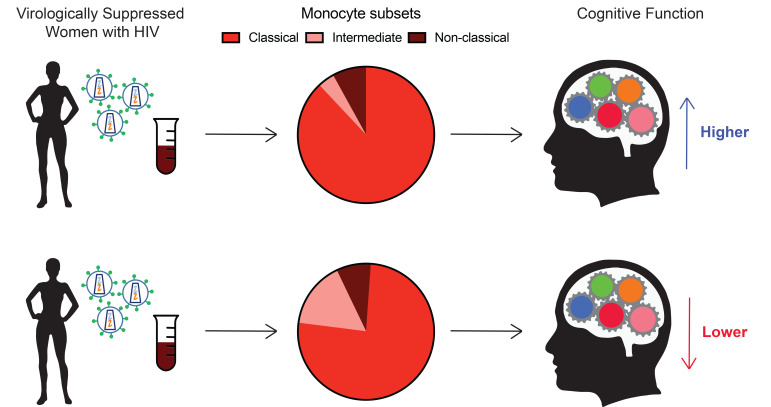

## Introduction

Neuropsychiatric complications persist despite effective antiretroviral therapy (ART) in people with HIV (PWH). Ongoing challenges remain in understanding the underlying pathophysiology and in identifying an easily measurable, quantitative, blood-based biomarker for predicting, detecting, and monitoring these complications. The role of monocytes in central nervous system (CNS) dysfunction is well supported by preclinical and clinical studies in HIV (1–3), yet no monocyte marker has been identified as a biomarker of neuropsychiatric dysfunction in PWH.

Monocytes are the initial defense against invading pathogens and play an essential role in the innate immune response. Monocytes are multifunctional and are either pro- or antiinflammatory mediators that guide the development of the innate and adaptive immune response to a pathogen (4, 5). In humans, monocytes can be identified using Toll-like receptor 2 (TLR2) as a surface marker (6, 7) and then divided into 3 circulating subsets, classified based on the expression levels of the surface proteins CD14 (coreceptor for Toll-like receptor 4) and CD16 (Fcγ receptor IIIa). Classical monocytes (CD14^+^CD16^–^) are the most abundant, composing approximately 80%–95% of all monocytes (8). These cells are effective phagocytes with multiple functions, including the coordination of innate immune responses, production of pro- and antiinflammatory cytokines, and migration into tissues in response to inflammatory signals (9, 10). Intermediate monocytes (CD14^+^CD16^+^) account for 2%–11% (8) of monocytes and are also called proinflammatory monocytes. This subset presents antigen (11); secretes high levels of proinflammatory cytokines (12); expresses chemokine receptors that drive their migration into tissues (13); and can expand in circulation following cardiac events, cancer, autoimmune disease, and stroke and in a number of bacterial and viral infections, including HIV (14, 15). Intermediate monocytes are also permissive to HIV infection because of high levels of CCR5 expression on the cell surface (16, 17). Nonclassical monocytes (CD14^–^CD16^+^), also known as patrolling monocytes, constitute 2%–8% of the monocyte population (8). This subset can survey the vasculature, patrol the endothelium (9), and promote homeostasis (18, 19). These cells rarely transmigrate into tissues in response to the inflammatory signals that drive classical and some intermediate monocytes (20). Similar to their intermediate precursors, this subset can also be infected with HIV and are increased during a variety of infections (16).

There is strong evidence that alterations in monocytes may contribute to neuropsychiatric complications in PWH. Increased circulation of intermediate monocytes (CD14^+^CD16^+^) infected with HIV is associated with immune activation and cognitive impairment in cross-sectional studies (2, 21–24). These monocytes are thought to transport HIV into the brain (3, 25–28), where HIV proteins, cytokines, and chemokines damage cells and tissue. Additionally, increases in peripheral soluble CD14 and CD163 remain markers commonly associated with CNS injury, specifically with cognitive impairment (1–3, 29). Other CNS outcomes, including mental health factors such as depression and posttraumatic stress, have yet to be linked to monocyte subsets in PWH. There is evidence in HIV-uninfected individuals that higher levels of intermediate monocytes can be used to differentiate between people exhibiting depressive symptoms and those individuals not exhibiting these symptoms (30). Further, the presence of monocyte subsets can be used in conjunction with other markers (e.g., C-reactive protein) to distinguish subtypes of depression (30).

There are well-documented sex differences in monocyte phenotypes in HIV-uninfected populations; however, studies are limited in PWH. Studies report a lower proportion of nonclassical (CD14^–^CD16^+^) monocytes in healthy women compared with men (31). Moreover, after adjusting for age, monocytes from healthy women have different expression patterns of CD38, CD62L, and CD115, and plasma levels of CXCL10 and soluble CD163 (sCD163) are elevated while sCD14 is decreased compared with healthy men (32). In the context of disease, preclinical studies indicate that trafficking of monocytes to the site of inflammation is decreased in female compared with male mice in a model of acute inflammation (33). Additionally, women with systemic lupus erythematosus show increased monocyte activation compared with men with lupus (34). Given these sex differences, we focus here on women with HIV (WWH) because the monocyte-cognition associations may not be the same in women and men with HIV.

Currently there is no clinical biomarker to predict neuropsychiatric function (includes cognition and mental health) in virally suppressed WWH. In the present study, we examined associations between subtypes of monocytes, CD4^+^ T cells, CD8^+^ T cells, CD4/CD8 ratio, and neuropsychiatric function in WWH. We hypothesized that higher levels of intermediate monocytes would be associated with lower cognitive function (via a comprehensive neuropsychological test battery) and more mental health symptoms (depression, posttraumatic stress via questionnaires).

## Results

### Cohort characteristics.

The Baltimore cohort included 25 Black, non-Hispanic WWH between 46 and 64 years of age (mean = 55, SD = 5.5, Supplemental Table 1; supplemental material available online with this article; https://doi.org/10.1172/jci.insight.146215DS1). All women were on effective ART, of whom 80% had HIV RNA less than 20 copies/mL and 100% had HIV RNA less than 250 copies/mL. The majority of participants were on an integrase inhibitor–based regimen with 16 (64%) on second-generation integrase strand transfer inhibitors (INSTIs) and 7 (28%) on first-generation INSTIs: 10 (40%) dolutegravir, 6 (24%) bictegravir, 5 (20%) elvitegravir, 2 (8%) raltegravir. The median monocyte subset percentages were 73.3% classical (CD14^+^CD16^–^), 15.1% nonclassical (CD14^–^CD16^+^), and 8.9% intermediate (CD14^+^CD16^+^). Cognitively, 40% of participants had global cognitive impairment, with a global neuropsychological (NP) demographically adjusted *z* score less than 1 SD from the reference group: Women’s Interagency HIV Study (WIHS) HIV-uninfected women (35, 36). On average, WWH in the Baltimore cohort demonstrated the greatest difficulty in verbal learning, delayed free recall, and recognition (demographically adjusted *z* scores < 1). A second cohort, the Bronx study cohort, was used to validate our findings in the Baltimore cohort. The Bronx study cohort was a predictive cohort as monocyte percentages were measured a 1 year (median) prior to NP assessment. These data were used to determine if monocyte proportions may predict future NP function in WWH. The Bronx cohort included 18 Black, non-Hispanic WWH between 32 and 58 years of age (mean = 48.5, SD = 8.3, Supplemental Table 1). All women were on effective ART, of whom 83% had HIV RNA less than 20 copies/mL and 100% had HIV RNA less than 250 copies/mL. The majority of participants were on tenofovir + emtricitabine (72%); ritonovir was used in 7 (39%) and atazanavir in 4 (22%). The median monocyte subset percentages were 72% classical (CD14^+^CD16^–^), 17% nonclassical (CD14^–^CD16^+^), and 11% intermediate (CD14^+^CD16^+^).

The demographics, behavior, and cellular composition in both cohorts were similar. However, the Baltimore cohort demonstrated greater cognitive difficulties compared with the Bronx cohort. Only 2 women (11%) in the Bronx cohort had a global NP demographically adjusted *z* score less than 1 SD from the reference group (WIHS HIV-uninfected women, refs. 35, 36), meaning 2 out of 18 women were globally impaired whereas 10 out of 25 women in the Baltimore cohort were globally impaired. On average, *z* scores for all NP outcomes did not fall below the 1 SD cutoff. The most difficult test on average for the participants in the Bronx cohort was the LNS working memory (*z* = –0.56), whereas for the Baltimore cohort it was delayed free recall and recognition (demographically adjusted *z* scores < 1). In the case of both cohorts, sociodemographic (e.g., age, education), behavioral (e.g., substance use, smoking), and clinical factors (e.g., CD4^+^ count) were not related to biomarkers (monocytes or T cells) and NP outcomes and thus were not confounders of the associations of interest. Full characteristics of the participants from both cohorts, including education, mental health, and drug use, are available in Supplemental Table 1.

### A larger proportion of mononuclear cell monocytes is associated with better cognitive function.

**Figure 1:**
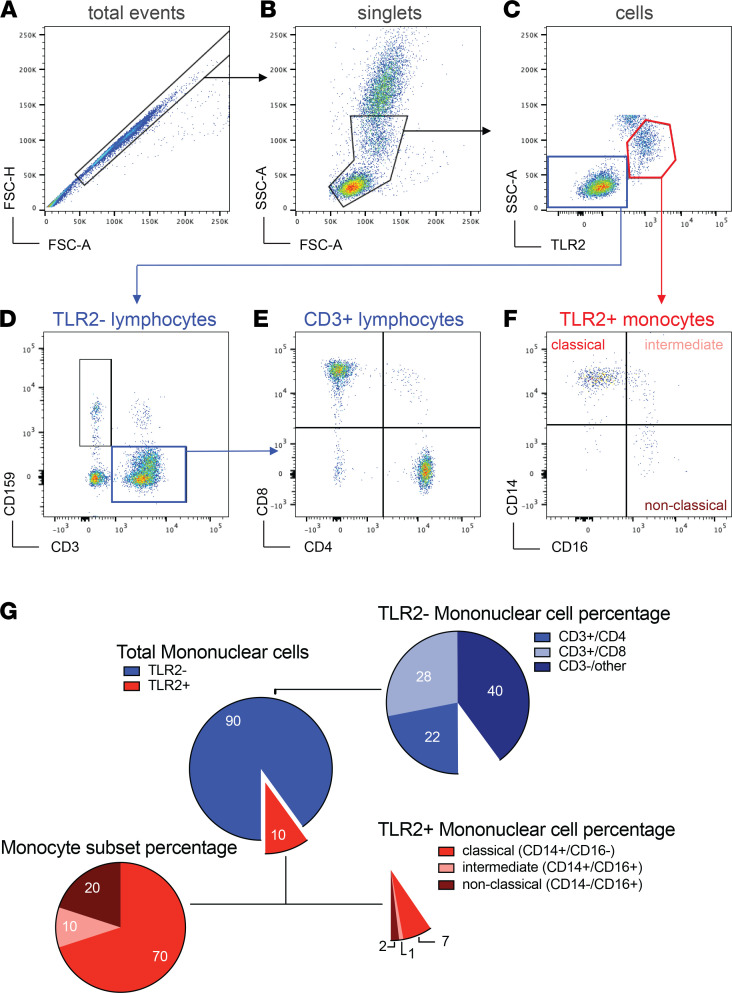
Monocyte subset and MNC calculation for Baltimore cohort. (**A**–**F**) A representative FACS gating scheme. (**A**) Doublets were excluded using FSC-A and FSC-H measurements, and (**B**) debris was gated out by drawing a gate on cell-sized events using FSC-A and SSC-A. (**C**) TLR2^+^ cells were gated as monocytes, and nongranulocyte TLR2^–^ cells were gated as lymphocytes. (**D**) TLR2^–^ lymphocytes were gated as CD3^+^ T cells or CD159a^+^ NK cells. (**E**) CD3^+^ cells were then gated as CD4^+^ or CD8^+^ T cells. (**F**) TLR2^+^ monocytes were gated based on the expression of CD14 and CD16 and classified as classical (CD14^+^CD16^–^), intermediate (CD14^+^CD16^+^), or nonclassical (CD14^–^CD16^+^) monocytes. (**G**) MNCs were counted in the whole blood sample, by summing both TLR2^+^ monocyte gating, which separates monocytes from granulocytes, and TLR2^–^ lymphocytes as smaller, agranular lymphocytes. Both were gated after debris and doublet removal. TLR2^+^ monocytes and CD4^+^ and CD8^+^ T cell populations were expressed as a percentage of MNCs. Monocyte subsets were expressed as either percentage of TLR2^+^ or percentage of MNCs.

**Figure 2:**
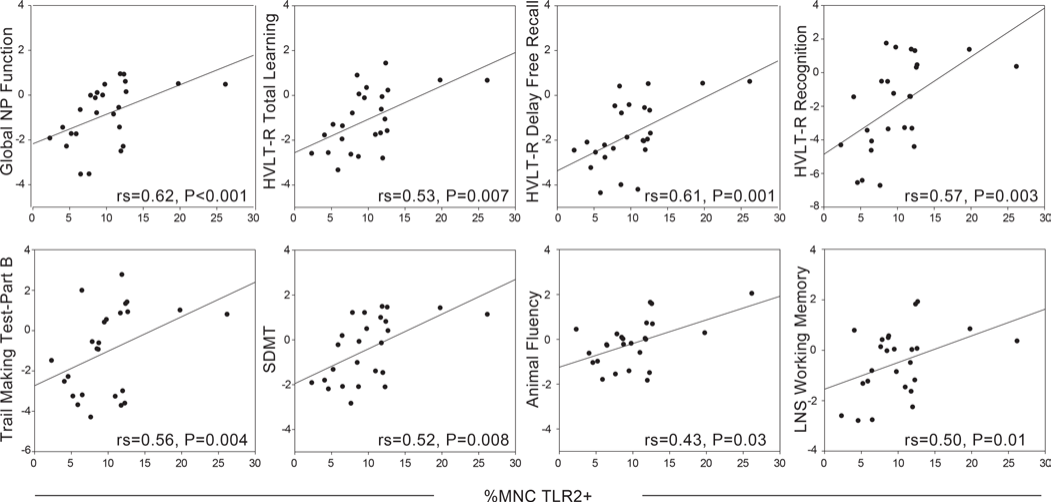
A higher percentage of TLR2^+^ MNCs is associated with higher cognitive function. Percentage of TLR2^+^ MNCs was measured concurrently with cognitive function in WWH (*n* = 25) living in Baltimore, Maryland, USA; Stroop-3 not shown (rs = 0.53, *P* < 0.001). Spearman’s Rho (rs) was used to examine the associations.

To assess whether the proportions of mononuclear cells (MNCs) in whole blood are associated with neuropsychiatric outcomes, we obtained whole blood samples in conjunction with NP testing and mental health measures from participants in the Baltimore cohort. Whole blood samples were analyzed by FACS to assess the proportion of MNCs, including monocytes, CD4^+^ T cells, and CD8^+^ T cells (Figure 1, A–F). MNCs were defined as the sum of TLR2^+^ monocytes and TLR2^–^ lymphocytes, which estimates the total proportion of mononuclear cells in a whole blood sample (Figure 1G). TLR2 was used as a marker for monocytes because it separates monocytes more definitively than FSC and SSC alone. This marker is ubiquitously expressed on human and macaque monocytes, and the percentage of monocytes expressing TLR2 does not change with SIV or HIV infection status (6) (Supplemental Figure 1). However, TLR2 is also expressed on other cell types, and therefore it is necessary to use both TLR2 and SSC to cleanly gate out this population. Only monocytes expressing TLR2 were included in our analysis. Any T cells or granulocytes that expressed low levels of TLR2 were removed via SSC gating (Supplemental Figure 2). TLR2^+^ monocytes were negative for CD3, CD8, CD159a, and CD20 and showed dim expression of CD4 as expected in human monocytes (37). Additionally, it is important to note, TLR2 was used as a phenotyping marker similar to CD14 and CD16, and not used to assess immune function, similar to how CD14 (LPS receptor) and CD16 (FC receptor) were not used to assess LPS and antibody signaling in these cells. A higher proportion of MNC monocytes (TLR2^+^) was associated with higher performance on all Hopkins Verbal Learning Test-Revised (HVLT-R) outcomes (total learning, delayed free recall, recognition), Symbol Digit Modalities Test (SDMT), Trail Making Test (TMT-Part B), Stroop Test Trial 3 (Stroop-3), Letter-Number Sequencing (LNS) working memory, and animal fluency (*P*s < 0.05; Figure 2 and Table 1). In contrast, the proportions of MNC CD4^+^ and CD8^+^ T cells, or CD4/CD8 ratio, were not associated with any of the NP or mental health outcomes (*P*s > 0.09; Supplemental Table 2).

**Figure 3:**
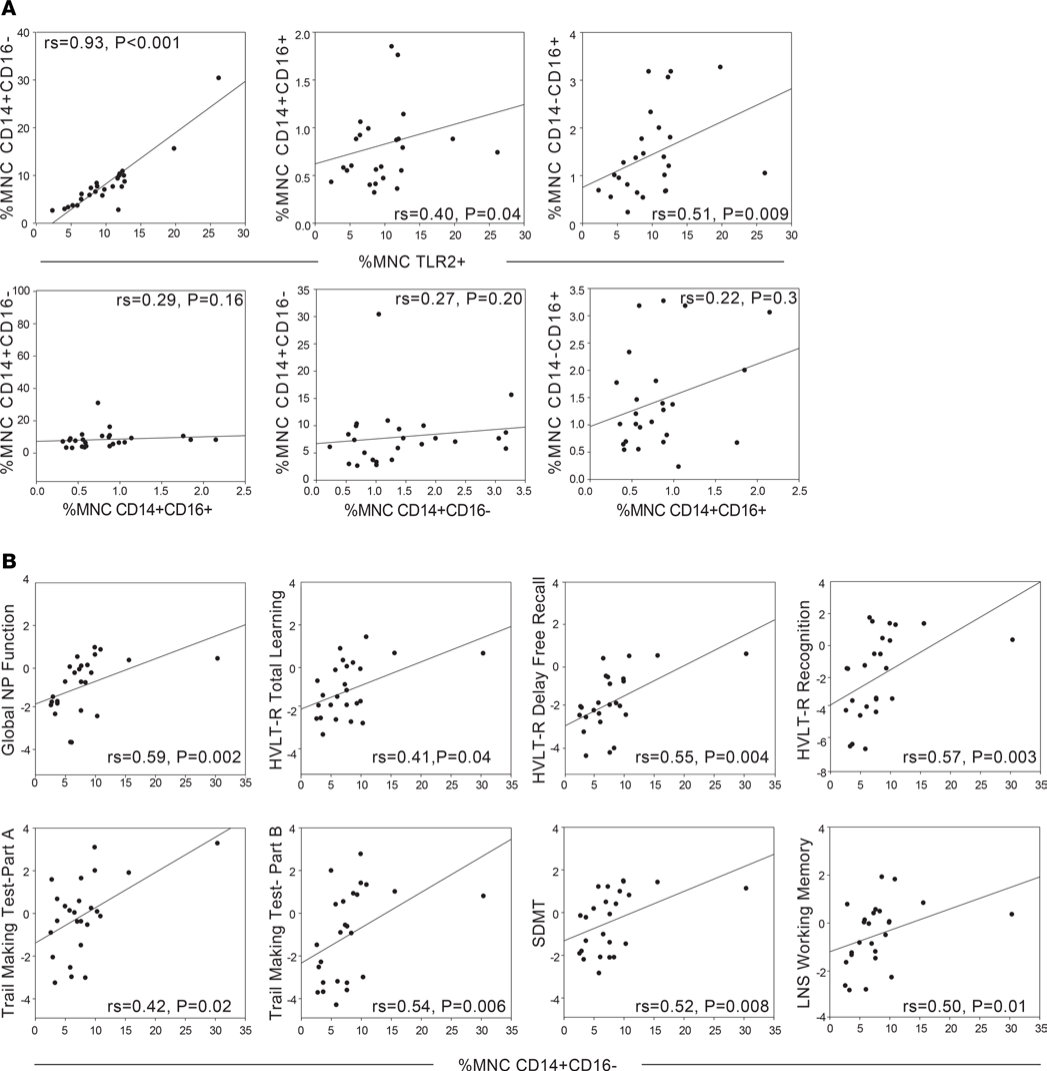
TLR2 numbers and associations with cognitive function are driven by an increase in classical monocytes, not maturation into other subsets. (**A**) Associations (using rs) between percentage MNCs TLR2^+^ and percentage MNC monocyte subsets in WWH living in Baltimore, Maryland, USA (*n* = 25). (**B**) Statistically significant associations (using rs) between cognitive function in WWH living in Baltimore, Maryland, USA (*n* = 25), and concurrent measurements of the percentage MNC classical monocyte subset; letter fluency (rs = 0.43, *P* < 0.05), animal fluency (rs = 0.49, *P* < 0.05), and Stroop-3 (rs = 0.47, *P* < 0.05) not shown. Classical (CD14^+^CD16^–^); intermediate (CD14^+^CD16^+^); nonclassical (CD14^–^CD16^+^).

To better understand the associations between total monocytes and NP function, we conducted a series of correlations in the Baltimore cohort focusing on (a) how the proportions of MNC monocyte subsets (classical, intermediate, and nonclassical) correlate with each other and MNC TLR2^+^ monocytes (total monocytes) and (b) how the proportions of MNC monocyte subtypes correlate with NP outcomes. When correlating the proportion of each MNC monocyte subset to the proportion of MNC TLR2^+^ monocytes, a higher number of classical monocytes correlated with a higher proportion of TLR2^+^ cells (rs = 0.93, *P* < 0.001; Figure 3A and Supplemental Table 3). There were no significant associations between the proportions of MNC monocyte subsets (*P*s > 0.16). This suggests that as the proportion of TLR2^+^ monocytes increases in blood, there is either an increase of classical monocytes egressing from the bone marrow or a lack of classical monocytes trafficking into the tissues in response to an inflammatory signal. When correlating the proportion of MNC monocyte subsets with NP outcomes, there were positive associations observed with the classical monocyte subset that closely mirrored the associations observed with MNC TLR2^+^ cells. A higher proportion of MNC classical monocytes was associated with higher global NP function and higher performance on all HVLT-R outcomes (total learning, delayed free recall, recognition), TMT-Part A and -Part B, SDMT, letter and animal fluency, Stroop-3, and LNS working memory (*P*s < 0.05; Figure 3B and Table 1). These data provide additional evidence that the positive outcomes observed with higher proportions of TLR2^+^ MNCs in whole blood are primarily driven by the classical monocyte fraction.

### A higher percentage of the intermediate monocyte subset is associated with worse cognitive function.

Assessing neuropsychiatric outcomes in relation to the proportions of all MNCs elucidates whether the proportion of each cell type compared with all cells is related to these outcomes. However, this type of analysis can minimize the contribution of small cellular populations, such as intermediate and nonclassical monocytes, which often make up less than 1% of all MNCs. To compensate for this possibility, we completed an alternate analysis that assessed neuropsychiatric outcomes in relation to the percentage of each monocyte subset within the monocyte cellular fraction alone (Figure 1G). This is an important comparison as assessing the percentage of monocyte subsets, using CD14/CD16 expression to distinguish cell types, is the most common way of analyzing these cells and would allow for comparisons between multiple cohorts. Additionally, it is important to know if absolute monocyte numbers are associated with cognitive function or if the percentages of monocyte subsets, within the monocyte system, are reflective of NP performance.

**Figure 4:**
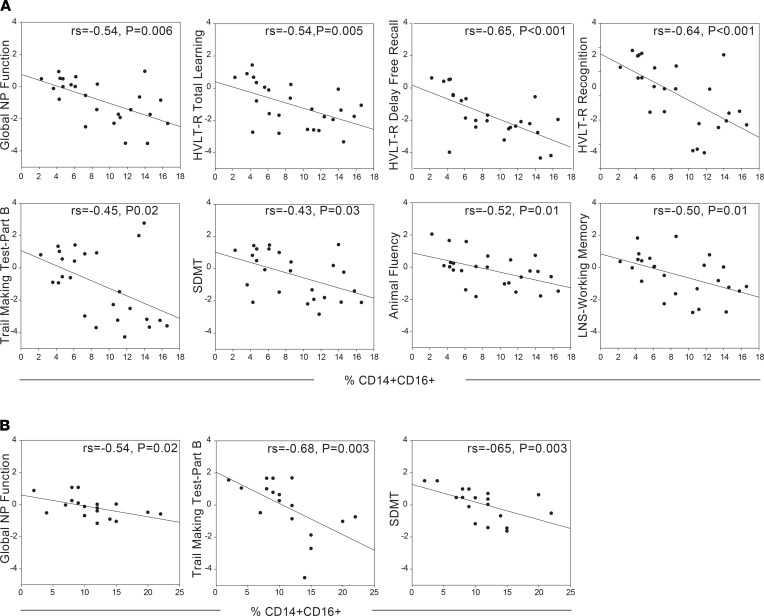
The percentage of intermediate monocyte subset negatively correlates with concurrent and predictive cognitive function. (**A**) Higher numbers of intermediate monocytes are associated (using rs) with lower cognitive function measured concurrently in WWH (*n* = 25) living in Baltimore, Maryland, USA. Grooved Pegboard dominant hand not shown (rs = –0.44, *P* < 0.05). (**B**) Higher numbers of intermediate monocytes are associated (using rs) with lower executive function and processing speed when cognition was assessed approximately 1 year following monocyte assessment (median = 1.14; IQR 0.58) in WWH living in Bronx, New York, New York, USA (*n* = 18).

When assessing the percentages of TLR2^+^ monocyte subsets (classical, intermediate, nonclassical) within the Baltimore cohort, a higher percentage of intermediate monocytes (CD14^+^CD16^+^) was associated with lower global NP function (rs = –0.54, *P* = 0.006; Figure 4A and Table 1). When examining each of the NP outcomes separately, a higher percentage of intermediate monocytes was also associated with lower performance on all outcomes on the HVLT-R (total learning, delayed free recall, recognition), TMT-Part B, SDMT, animal fluency, LNS working memory condition, and Grooved Pegboard dominant hand (*P*s < 0.05). However, the percentage of nonclassical monocytes was not associated with any NP outcomes (*P*s > 0.12). Thus, when all CD16^+^ monocytes (intermediate + nonclassical) were combined, as has been reported in previous analyses (2, 3, 22–24, 27, 28, 38), it resulted in no significant associations with NP outcomes, except for TMT-Part B (rs = –0.44, *P* = 0.02) and animal fluency (rs = –0.49, *P* = 0.01; Supplemental Table 4). Although the percentage of classical monocytes was not associated with global NP function (*P* = 0.11), a higher percentage was associated with higher performance on TMT-Part B (rs = 0.44, *P* = 0.03) and animal fluency (rs = 0.54, *P* = 0.006). Mental health measures were not significantly associated with any of the monocyte subsets (*P*s > 0.27).

To confirm our findings, we assessed monocyte subsets in relation to NP and mental health outcomes in a secondary validation and predictive cohort. In the Bronx cohort, monocyte subset percentages were assessed in PBMCs and neuropsychiatric assessments performed approximately 1 year later (median). In this cohort, a higher percentage of intermediate monocytes was also associated with lower global NP function (rs = –0.54, *P* = 0.02; Figure 4B and Table 2). When examining each of the NP outcomes separately, a higher percentage of intermediate monocytes was also associated with lower performance on TMT-Part B (rs = –0.68, *P* = 0.003) and SDMT (rs = –0.65, *P* = 0.003). Similar to the Baltimore cohort, classical and nonclassical monocytes were not associated with global NP function in the Bronx cohort (*P*s > 0.65). There were no associations between the percentage of classical or nonclassical monocytes and any NP outcome (*P*s > 0.14). Mental health measures were not significantly associated with any of the monocyte subsets (*P*s > 0.11). These data suggest that the percentages of the intermediate monocytes within the monocyte cellular fraction are reflective of worse cognition both concurrently and over time while classical monocytes do not appear to play a role long term.

### The proportion of MNC monocytes (particularly classical) and the percentage of intermediate monocyte subset were associated with cognitive function even among virally suppressed subjects.

To ensure that our pattern of significant associations was not being driven by the few participants with low levels of viremia (HIV RNA < 250 copies/mL), we reran our correlations after removing these individuals. After removing the 5 participants with HIV RNA between 20 and 238 copies/mL in our Baltimore cohort, we observed the same pattern of associations between %MNC TLR2^+^, classical monocytes (%MNC and %subsets), and cognitive function as we did in the full cohort (Supplemental Table 5). The same pattern of associations remained between the percentage of intermediate monocyte subsets and cognitive outcomes except for the Grooved Pegboard dominant hand. We also reran our analyses in the Bronx cohort, and again no change was observed in the pattern of associations after removing the 3 women with HIV RNA less than 100 copies/mL (data not shown). These data suggest that low-level viral blips are not the driving force of the monocyte subset associations with cognitive function observed in the virally suppressed WWH.

## Discussion

In this study, we identified a cognitive biomarker that can be readily measured in blood and may be used to assess neuropsychiatric function in WWH. Here, we examined associations between total monocytes, monocyte subsets, T cells, and neuropsychiatric function in WWH. First, in 2 independent cohorts, higher proportions of intermediate monocytes (CD14^+^CD16^+^) were associated with lower global cognitive function as well as lower executive function (mental flexibility) and processing speed. These associations were present when assessing monocytes concurrently with (Baltimore cohort), and approximately 1 year before (Bronx cohort), the NP assessment. Our analysis indicated that the percentage of intermediate monocytes within the monocyte fraction of cells, and not the proportion of MNC intermediate monocytes, was associated with lower cognitive function. Second, when assessing monocytes and NP function concurrently, there were a number of other cognitive correlates with intermediate monocytes, including those that assess verbal learning and memory, semantic fluency, working memory, and motor function. Third, the opposite patterns of associations were observed when examining associations between total MNC monocytes, classical monocytes (subset percentage and MNC proportion), and cognitive function; a greater number of total and classical monocytes was associated with better cognitive function. Fourth, there were no significant associations between monocytes and mental health outcomes and, as expected, no associations between CD4^+^ and CD8^+^ T cells and CD4/CD8 ratio and cognitive function. Fifth, small detectable viral blips (20–250 copies/mL) did not drive the observed associations with cognitive function because we found that removal of participants with detectable viral blips primarily strengthened the pattern of associations. Overall, our findings suggest that the proportion of the intermediate monocyte subset in blood provides insight into immune-brain connections, specifically cognitive function in virally suppressed WWH.

It is widely accepted that lentiviral infection of the CNS targets cells of monocyte-macrophage-microglial lineage, causing immune activation and blood-brain barrier disruption (2, 16, 29). The association of these events with increases in monocyte migration into brain and the emergence of intermediate monocytes (CD14^+^CD16^+^) in the blood has been previously been reported (3, 22, 23). However, the proportion of intermediate monocytes in blood of virally suppressed WWH has never been directly associated with cognitive function to our knowledge. Currently, markers of chronic immune activation, such as circulating monocytes, show the most promise as blood biomarkers to measure NP function during viral suppression. Circulation of intermediate monocytes infected with HIV contributes to chronic immune activation, and these monocytes are thought to transport HIV into the brain (25), where HIV proteins, cytokines, and chemokines damage cells and tissue. While numerous studies demonstrate that higher levels of monocyte inflammatory markers (sCD163, sCD14) in blood are associated with lower NP function in PWH (39–43), including women (41, 42), no study to date to our knowledge has demonstrated links between specific monocyte subsets and global or domain-specific NP function in virally suppressed WWH. Previous studies assessing monocyte subsets in relation to cognitive function have combined subsets (e.g., all CD16^+^ cells) (2, 3, 22–24, 27, 28, 38), focused on markers on monocyte cells (e.g., CCR2, CCR5, CD38, CD163) (24, 44, 45), or measured peripheral proinflammatory proteins (e.g., sCD163, sCD14) (41, 43) as a surrogate. One study assessed HIV DNA levels within monocyte subsets and correlated DNA levels with cognition; however, correlations with the actual monocyte subsets and cognition were not included (46). An additional study assessed HIV DNA levels in PBMCs and found a correlation with CCR2 expression on intermediate monocytes and HIV-associated neurocognitive disorder (HAND) (24).

Our study focused directly on the proportion of monocyte subsets as a biomarker rather than downstream indicators. In addition to CD14 and CD16, we have used TLR2 as a marker to more specifically separate monocytes from NK cells and granulocytes (6, 7). Including TLR2 as a marker provides a more accurate assessment of total monocytes and proportions of monocyte subsets. Additionally, we analyzed each monocyte subset independently, as each subset provides information about inflammation and homeostasis in the host while grouping monocyte subsets together obscures the function of each subset. Recent studies have shown that the intermediate and nonclassical monocytes are transcriptionally and functionally distinct (47) and that the intermediate monocytes are the cells that preferentially migrate across the blood-brain barrier (3, 27). In fact, if we combine both of the CD16^+^ monocyte subsets (intermediate and nonclassical monocytes) in our data set, the previously observed associations between monocytes and cognition are lost except for animal fluency and TMT-Part B. Finally, the use of fresh samples, as was done with both cohorts, allows us to account for all monocytes present in the blood at the time of draw. Freezing and subsequent thawing of PBMCs can lead to preferential loss of inflammatory cell types (48). Together, these alternative methods of acquisition and monocyte subset analysis may explain why we observed a strong phenotype with intermediate monocyte subset percentages and cognition that has been previously overlooked in other studies.

Our findings provide evidence that the percentage of the intermediate monocyte subset is associated with cognitive function in WWH. We demonstrate that a greater proportion of total TLR2^+^ monocytes present in blood is associated with better concurrent cognition whereby the magnitude of associations across all NP outcomes was similar, suggesting a general rather than domain-specific association with cognitive function. Additionally, higher total TLR2^+^ monocytes directly correlated with an increase in the classical monocyte cell type, as expected given that classical monocytes typically compose 80%–95% of total monocytes in the blood (8). A larger proportion of classical monocytes is indicative of homeostasis and low levels of inflammation, as classical monocytes are relatively immature in the blood and do not mature until they receive inflammatory signals that recruit them to injured tissues (49, 50). Therefore, a higher proportion in the blood suggests a lack of recruitment to tissues and lower inflammation. In HIV specifically, classical monocyte levels are lower in ART-suppressed PWH compared with HIV-uninfected individuals (51, 52). Therefore, it is possible that the WWH in our cohort with a higher proportion of classical monocytes are more similar to healthy individuals and thus associated with better cognitive function.

In contrast, the percentage of the intermediate monocyte subset significantly correlated with impaired concurrent (global NP function, *P* < 0.001) and predictive (global NP function, *P* < 0.05) cognitive function. Additionally, intermediate monocytes did not strongly correlate with total TLR2^+^ monocytes or classical monocytes. This suggests that an increase in the percentage of the intermediate monocyte subset in the blood is a result of (a) classical monocytes trafficking out of blood into tissue, altering the proportions of cells in the blood, or (b) a greater number of classical monocytes maturing into intermediate monocytes, not an increase in total monocytes in blood. Further, the proportion of intermediate monocytes to all MNCs in the blood is not associated with cognitive function. This suggests that it is not the absolute number of intermediate monocytes that is important but the proportion of intermediate monocytes to the other monocyte subsets. However, it is also possible that the MNC analysis may mask the effects of very small cell populations. Intermediate monocytes have a higher capacity to secrete cytokines in the blood and are reflective of an inflammatory environment (49, 50). Therefore, a change in their proportion within the monocyte subsets would likely be associated with a change in inflammation in the host. Additionally, when comparing monocyte subset proportions between ART-suppressed PWH and HIV-uninfected individuals, it has been shown that intermediate monocyte levels are the same while classical and nonclassical monocyte proportions differ between the 2 groups (51, 52). This reinforces the conclusion that the subset percentage, and not the absolute number, of intermediate monocytes is important for cognitive function.

When assessing monocytes and cognition concurrently, the strongest NP outcomes that correlated with intermediate monocytes included the HVLT-R, animal fluency, TMT-Part B, SDMT, and LNS working memory condition. The deficits observed in these tasks most likely relate to changes in brain function as chronic, low-level inflammation in PWH may lead to the migration of intermediate monocytes into corticolimbic brain regions necessary to perform these NP tests (53–57). When we assessed monocytes approximately 1 year before NP testing, the 2 NP tests that remained significant included TMT-Part B and SDMT. Functional neuroimaging studies of the NP tasks that are significant in both cohorts implicate the prefrontal cortex as being required to complete each of these tasks (53, 54). Therefore, elevated intermediate monocytes may be predictive of prefrontal function throughout HIV infection despite ART suppression.

Despite the small sample size, both cohorts were acquired, processed, and analyzed independently, and therefore the reproducibility of our concurrent and predictive findings gives weight to the associations observed. In fact, the strength of the correlations observed, despite the small sample size in each cohort, is suggestive of an important and relevant finding. The generalizability of our findings may be limited given the cohorts are composed of predominately low-income women of color with HIV. However, this is an important and relevant HIV population, given that people of color compose the majority of people with HIV infections in the United States and around the world. Studies incorporating men and healthy individuals would determine the generalizability of our findings. We are not suggesting that this finding is specific to WWH as previous work has shown that the percentages of CD16^+^ monocytes in blood of men and women with HIV do not differ (28), suggesting that these cells are present in men at a similar frequency and may relate to cognition in this population as well. Additionally, it should be noted that in this study women were defined by self-report biological sex, and the terms woman and female are used interchangeably in this study. This is the first study to our knowledge to directly assess intermediate monocyte percentages in blood and cognition in any virally suppressed group living with HIV and will need expansion. The effect of ART regimens on monocyte subset and/or cognition was not addressed in this study due to small sample sizes and the relatively heterogeneous treatment regimens within each cohort. Finally, our findings focus on cognition and HIV; however, it is likely these findings will extend beyond HIV and contribute to other neurological diseases and disorders because CD14^+^CD16^+^ monocytes have emerged as a principal driving cell type for a number of proinflammatory conditions.

This study has provided evidence of a cognitive biomarker that can be readily measured in blood in WWH. We report that the percentage of the intermediate monocyte subset significantly correlates with impaired cognitive function and that the levels of intermediate monocytes may have a long-lasting effect on cognition in WWH. These findings warrant future larger scale studies to identify the optimal cut point for the percentage of intermediate monocytes that predict cognitive impairment in PWH.

## Methods

### Study cohorts and procedures.

The initial study cohort (Baltimore cohort) included 25 WWH enrolled in a phase 0 clinical trial between December 5, 2018, and March 3, 2020, focused on the effects of glucocorticoids on cognition (https://clinicaltrials.gov/ct2/show/NCT03237689; R01-MH113512). These participants passed a phone screen and were scheduled for their study enrollment visit, which consisted of informed consent, completing an NP test battery, questionnaires (including demographic and mental health screeners), a urine toxicology screen, and a blood draw. The enrollment visit occurred prior to study randomization. Initial inclusion criteria were ages 18 to 65, female, living with HIV, English as a first language, and able to give informed consent and travel to the study site for study participation. Exclusion criteria were current use of hormone-based contraceptives; currently pregnant, postpartum, or lactating; current regular use of steroids, closed head injury, history of schizophrenia or schizoaffective disorder, current untreated hypertension or diabetes, history of dementia or any other neurologic CNS or AIDS-defining disorder, substance use disorder in the past 6 months, and positive urine toxicology screen (except marijuana) or breathalyzer and/or any evidence of acute intoxication or withdrawal. All Baltimore cohort samples were fresh, never frozen, whole blood samples. The validation study cohort (Bronx cohort) included 18 WWH over the age of 18 who enrolled in a previous published study at the Bronx WIHS site between June 25, 2012, and October 4, 2013, that focused on monocytes and HAND (23). These participants completed the same NP battery, questionnaires, urine toxicology screen, and blood draw. Exclusion criteria included any crack, cocaine, and/or heroin use in the past 6 months or hepatitis C antibody positive. While the blood draw and NP test battery were done concurrently in the Baltimore cohort, the Bronx cohort’s NP testing and mental health screeners were completed 0 to 1.55 years later (median = 1.14, IQR = 0.58). All Bronx cohort samples were fresh, never frozen, PBMCs.

### NP test battery.

Both cohorts of women completed the same NP test battery. The NP test battery included the HVLT-R (outcomes = total learning, delay free recall, recognition), the LNS (outcomes = experimental [working memory] and control [attention] conditions total correct), the TMT (outcomes = time to complete Parts A and B), the Stroop-3 (outcome = time to complete trials 1 [color reading], 2 [color naming], and 3 [color-word]), the SDMT (outcome = total correct), letter-guided verbal fluency (Controlled Oral Word Associations Test; outcome = total correct words generated across 3 trials [F, A, S]), animal fluency (outcome = total correct animals generated), and the Grooved Pegboard (outcomes = time to completion, dominant and nondominant hand). Demographically adjusted *z* scores were calculated for each outcome using data from HIV-uninfected WIHS women (35, 36). Global NP function was calculated as the average of the demographically adjusted *z* scores for each individual outcome measure.

### Mental health screeners.

Both cohorts of women completed the (a) CES-D, which assesses depressive symptoms (58); (b) PSS-10, which measures the degree of uncontrollability, unpredictability, and overload in the respondent’s life (59, 60); and (c) PCL-C, which assesses posttraumatic symptom burden (61). Higher scores on each of these measures indicate more symptomatology.

### FACS.

For the Baltimore cohort, whole blood samples were stained with pretitered amounts of monoclonal antibodies using 100 μL of whole blood at room temperature for 20 minutes. The antibody panels consisted of anti-CD3 V500 (clone SP34-2; BD Biosciences), anti-CD4 PerCP-Cy5.5 (clone L200, BD Biosciences), anti-CD8a BV570 (clone RPA-T8; BioLegend), anti-CD159a APC (clone NKG2A; Beckman Coulter), anti-TLR2 AF488 (clone 11G7; BD Biosciences), anti-CD14 BV650 (clone M5E2; BD Biosciences), and anti-CD16 AF700 (clone 3G8; BioLegend). Whole blood samples were then lysed and fixed in 2 mL of FACS Lysing Solution (BD Biosciences) for 10 minutes at room temperature. Samples were collected in a centrifuge at 400*g* for 5 minutes, washed in 2 mL of 1× phosphate-buffered saline (PBS), and then resuspended in 0.5 mL of PBS for analysis. Flow cytometry was performed on a BD LSRFortessa (BD Biosciences). Data were analyzed using FlowJo 10.0.8 software (FlowJo, LLC). See Figure 1, A–F, for gating scheme. MNCs were counted as agranulocytes in the whole blood sample, summing both TLR2^+^ monocyte gating, which separates monocytes from granulocytes, and TLR2^–^ lymphocytes as smaller, agranular lymphocytes. Both were gated after debris and doublet removal. TLR2^+^ monocytes and CD4^+^ and CD8^+^ T cell T cell populations were expressed as a percentage of MNCs. Monocyte subsets were expressed as either percentage of TLR2^+^ cells or percentage of MNCs (Figure 1G).

For the Bronx cohort, details on methods regarding cell isolation and monocyte identification by flow cytometry have been previously published (23). In brief, PBMCs were isolated by Ficoll density gradient centrifugation and the cells stained with a cocktail of fluorochrome-coupled monoclonal antibodies specific for human CD14 (clone M5E2), CD16 (clone 3G8), CD3 (clone HIT3a), CD19 (clone HIB19), CD56 (clone B159), CD66b (clone G10F5), and HLA-DR (clone G46-6), or corresponding isotype-matched, negative control antibodies (all antibodies were purchased from BD Biosciences; ref. 23). PBMCs (2–5 × 10^5^) were washed with calcium- and magnesium-free PBS (Gibco, Thermo Fisher Scientific), supplemented with 1% BSA (Thermo Fisher Scientific), and were incubated in the dark on ice for 30 minutes with the appropriate antibodies. Following staining, PBMCs were washed once with PBS/1% BSA and fixed with 0.2 mL 2% paraformaldehyde. Flow cytometry was performed on a BD FACSCanto II flow cytometer and data were analyzed using FlowJo. Monocytes were defined according to forward- and side-scatter characteristics and were identified as CD14- and HLA-DR–positive and CD3-, CD19-, CD56-, and CD66b-negative as described previously (23).

### Statistics.

A series of Spearman’s Rho correlations (rs) were conducted to examine associations between total monocytes, monocyte subsets, T cell populations, T cell ratios, and NP performance. Adjusted analyses were not necessary as the measured sociodemographic (e.g., age, education), behavioral (e.g., substance use, smoking), clinical factors (e.g., CD4^+^ count; Supplemental Table 1), and female-specific factors (menopausal stage) were not related to both biomarkers and NP performance. All analyses were conducted in IBM SPSS Statistics for Windows (Version 25.0, IBM Corp). Significance was set at *P* < 0.05.

### Study approval.

Data collection as part of the primary cohort (Baltimore) was approved by the Johns Hopkins University Institutional Review Board. Data collection as part of the validation cohort (Bronx) was approved by the Institutional Review Board at the Montefiore Medical Center, the Albert Einstein College of Medicine, and the Mount Sinai Program. Informed written consent was obtained from all participants prior to enrollment into each study site. The study was conducted according to Declaration of Helsinki principles.

## Author contributions

RTV and LHR conceived of and designed the study. RTV, DWW, ENS, and JEC designed experiments. DWW, ES, EAF, and CMA performed experiments. RTV, DWW, ENS, and LHR analyzed the data. JMC and TTB collected specimens. LHR, JEC, JWB, and KA provided materials. RTV and LHR wrote the initial drafts of the manuscript. All authors (RTV, DWW, ENS, CMA, EAF, JMC, TTB, PMM, KA, JWB, JEC, and LHR) provided critical review of the manuscript for important intellectual content and contributed to and approved the final manuscript.

## Supplementary Material

Supplemental data

Trial reporting checklists

ICMJE disclosure forms

## Figures and Tables

**Table 1:**
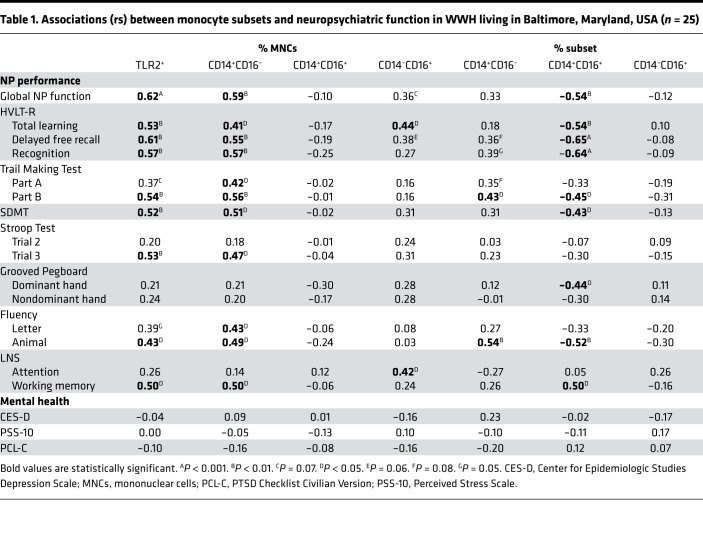
Associations (rs) between monocyte subsets and neuropsychiatric function in WWH living in Baltimore, Maryland, USA (*n* = 25)

**Table 2:**
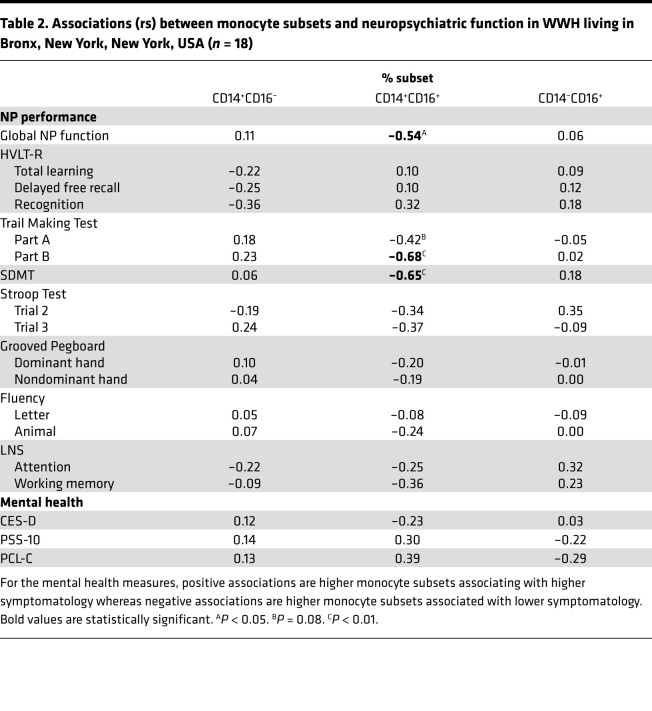
Associations (rs) between monocyte subsets and neuropsychiatric function in WWH living in Bronx, New York, New York, USA (*n* = 18)

## Version 1 04/29/2021

In-Press Preview

## Version 2 06/08/2021

Electronic publication
