# Supplementary material for: Higher circulating intermediate monocytes are associated with cognitive function in women with HIV
Source: JCI Insight. 2021 Jun 8;6(11):e146215. doi: 10.1172/jci.insight.146215 (PMC8262276; doi:10.1172/jci.insight.146215)
Supplement: Supplemental data [file jciinsight-6-146215-s054.pdf]

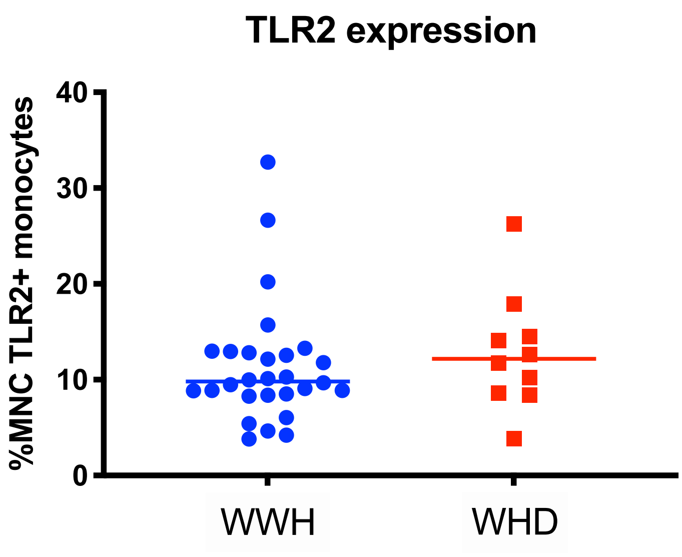

Supplemental Figure 1. TLR2 Expression in Women with HIV (WWH) and Women Healthy Donors (WHD). MNC TLR2% positive cells were compare between WWH (n=28) and HD (n=10) to determine if there was a difference in expression between the two groups that could have been attributed to HIV infection. There was no significant difference in TLR2 expression between the two populations.

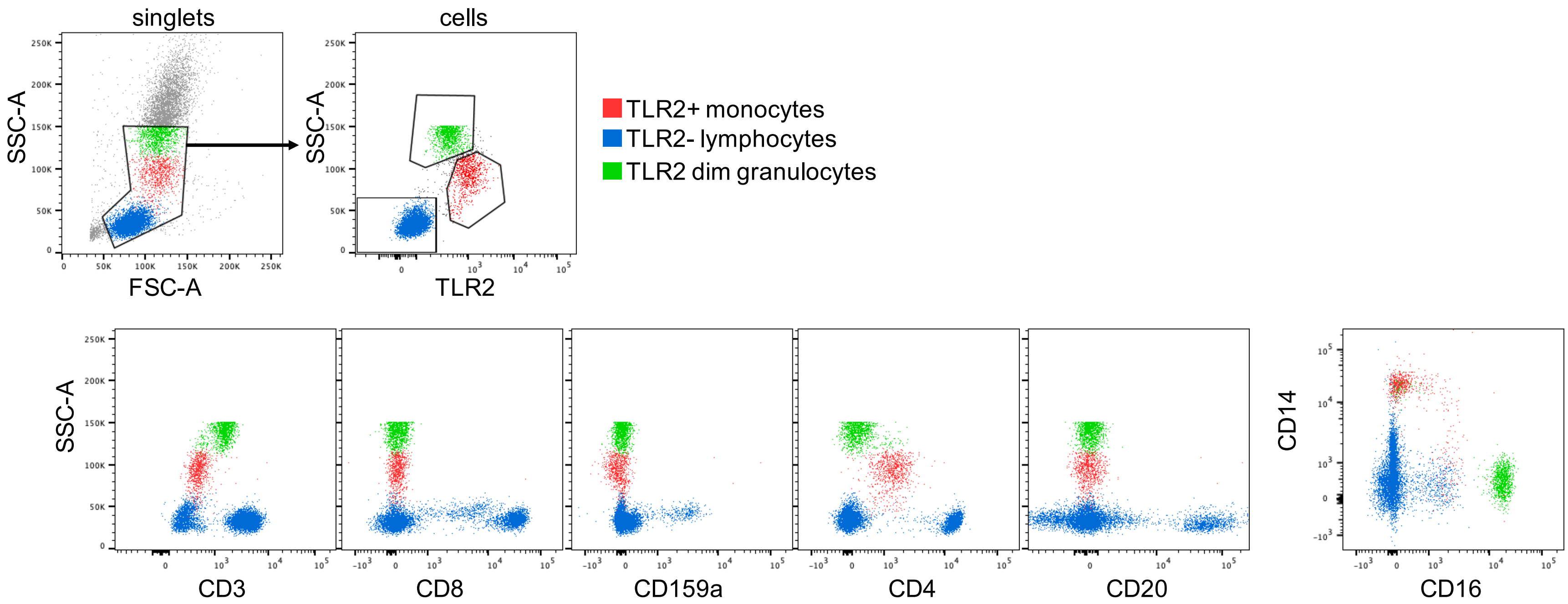

Supplemental Figure 2. Comparison of cell types present in human whole blood based on TLR2 expression and side scatter. Following removal of doublets (shown in Figure 1) cells were gated to remove debris and high granularity, large granulocytes. This population of cells was then gated on bright TLR2 expression (red) to refine the monocyte gate. These populations are further analyzed below, showing that the TLR2+ monocytes are negative for CD3, CD8, CD159a, and CD20, and show dim expression of CD4 as expected in human monocytes. The TLR2 dim granulocyte population is also negative for these common lymphocyte markers. The populations are compared in their expression of monocyte subtype markers CD14 and CD16. TLR2- lymphocytes are negative for CD14, and NK cells express CD16 at a similar level. TLR dim granulocytes express high levels of CD16 and no CD14. The red gated cells are the only cells used in the monocyte analysis.

Supplemental Table 1. Sociodemographic, behavioral and clinical factors as well mental health characteristics, and neuropsychological (NP) test function in the Baltimore cohort and Bronx validation and predictive cohort samples.

	**Baltimore, MD (n=25)**	**Bronx, NY (n=18)**
Sociodemographic factors		
Age, M (SD)	55.6 (5.5)	48.5 (8.3)
Years of education, M (SD)	11.6 (2.2)	11.0 (2.7)
WRAT-R reading subscale, M (SD)	54.8 (12.3)	84.4 (16.2)
Black, n (%)	24 (96)	11 (61)
Hispanic, n (%)	0 (0)	7 (38)
Behavioral and clinical factors		
Current smoker, n (%)	13 (52)	7 (39)
Recent use		
Marijuana, n (%)	4 (16)	3 (17)
Alcohol use, n (%)	6 (24)	6 (33)
Crack, cocaine, &/or heroine, n (%)	0 (0)	0 (0)
On effective ART, n (%)	25 (100)	18 (100)
HIV duration, median (IQR)	-	12.1 (9.8)
Years on ART, median (IQR)	-	14.7 (16)
CD4 count, median (IQR)	-	624 (325)
CD4 nadir, median (IQR)	-	235 (332)
HIV RNA		
Undetectable (<20 copies/ml)	20 (80)	15 (83)
Detectable (< 250 copies/ml)	25 (100)	18 (100)
Mental health measures, median (IQR)		
CES-D total score	12.8 (7.7)	11.6 (9.0)
PSS-10	9.3 (8.2)	12.8 (7.4)
PCL-C	27.1 (13.8)	32.7 (11.3)
NP performance, median (IQR)		
Global NP function	-0.83 (1.34)	-0.18 (0.75)
HVLT-R		
Total learning	-1.06 (1.35)	0.34 (1.63)
Delayed free recall	-1.73 (1.46)	0.23 (2.32)
Recognition	-1.95 (2.70)	-0.55 (2.58)
Trail Making Test		
Part A	-0.10 (1.79)	-0.28 (1.37)
Part B	-1.01 (2.13)	0.26 (2.25)
SDMT	-0.40 (1.42)	0.38 (1.58)
Stroop Test		
Trial 2	-0.56 (1.88)	-0.26 (2.43)
Trial 3	-1.47 (2.80)	-0.24 (1.08)
Grooved Pegboard		
Dominant	-1.63 (3.02)	0.36 (2.06)
Non-dominant hand	-1.49 (2.83)	0.51 (1.74)

Fluency		
Letter	0.35 (1.28)	-0.33 (1.98)
Animal	-0.18 (1.04)	-0.10 (1.63)
LNS		
Attention	-0.26 (1.09)	-0.15 (2.00)
Working Memory	-0.48 (1.32)	-0.56 (1.70)
%Markers, median (IQR)		
MNC CD4+ T cells	24.3 (8.2)	-
MNC CD8+ T cells	30.7 (9.6)	-
MNC TLR2+	10.0 (5.0)	-
MNC classical monocytes	7.9 (5.6)	-
MNC intermediate monocytes	0.8 (0.5)	-
MNC non-classical monocytes	1.4 (0.9)	-
Classical monocytes	73.3 (9.6)	72.5 (15)
Intermediate monocytes	8.9 (4.3)	11 (6)
Non-classical monocytes	15.1 (7.6)	17 (15)

Note. ART=antiretroviral therapy; CES-D=Center for Epidemiologic Studies Depression Scale; Heavy alcohol use is >7 drinks per week; HVLT-R=Hopkins Verbal Learning Test Revised; IQR=interquartile range; M=mean; MNC=mononuclear cells; PSS-10=Perceived Stress Scale; PCL-C=PTSD Checklist Civilian Version; SD=standard deviation; SDMT=Symbol Digit Modalities Test; WRAT-R=Wide Range Achievement Test-Revised.

Supplemental Table 2. Associations (using Spearman's Rho) between percent mononuclear cells (MNC) T cells and neuropsychiatric function in women with HIV living in Baltimore, MD (n=25).

	**Proportion MNC**		
NP performance	CD4+	CD8+	CD4:CD8
Global NP function	0.02	-0.06	0.02
HVLT-R			
Total learning	-0.04	-0.24	0.07
Delayed free recall	-0.04	-0.13	0.01
Recognition	0.18	-0.34[T]	0.27
Trail Making Test			
Part A	-0.05	0.14	-0.11
Part B	0.09	0.01	0.01
SDMT	0.11	-0.04	0.06
Stroop Test			
Trial 2	-0.07	0.19	-0.13
Trial 3	0.01	0.06	-0.06
Grooved Pegboard			
Dominant	-0.01	-0.16	0.10
Non-dominant hand	-0.10	-0.02	-0.05
Fluency			
Letter	-0.16	0.12	-0.10
Animal	0.24	-0.25	0.22
LNS			
Attention	-0.21	0.28	-0.26
Working Memory	-0.21	0.07	-0.13
Mental health			
CES-D	0.20	-0.03	0.15
PSS-10	-0.33	0.02	-0.22
PCL-C	-0.06	-0.01	0.01

Note. *P<0.05; [T]P=0.09; CES-D=Center for Epidemiologic Studies Depression Scale; HVLT-R=Hopkins Verbal Learning Test Revised; MNC=mononuclear cells; PSS-10=Perceived Stress Scale; PCL-C=PTSD Checklist Civilian Version; SDMT=Symbol Digit Modalities Test

Supplemental Table 3. Associations (Spearman's Rho) between percentage of mononuclear cells (MNC) TLR2+ and MNC monocyte subsets in women with HIV living in Baltimore, MD (n=25).

	**Proportion MNC**			
	TLR2+	CD14+CD16-	CD14+CD16+	CD14-CD16+
%MNC TLR2+	1.00	0.93***	0.41*	0.51**
%CD14+CD16-		1.00	0.29	0.26
%CD14+CD16+			1.00	0.22
%CD14-CD14+				1.00

***P<0.001; **P<0.01; *P<0.05

Supplemental Table 4. Associations (Spearman's Rho) between %CD16+ monocytes (intermediate+ non-classical) and neuropsychiatric function in women with HIV RNA undetectable (<20cp/ml) HIV living in Baltimore, MD (n=20).

	**Intermediate/non-classical subsets**
	%CD16+
NP performance	
Global NP function	-0.31
HVLT-R	
Total learning	-0.14
Delayed free recall	-0.35[‖]
Recognition	-0.38[†]
Trail Making Test	
Part A	-0.27
Part B	**-0.44***
SDMT	-0.27
Stroop Test	
Trial 2	0.05
Trial 3	-0.21
Grooved Pegboard	
Dominant	-0.08
Non-dominant hand	0.04
Fluency	
Letter	-0.24
Animal	**-0.49****
LNS	
Attention	0.22
Working Memory	-0.34[T]
Mental health	
CES-D	-0.21
PSS-10	0.15
PCL-C	0.16

**P<0.01; *P<0.05; [†]P=0.06; [‖]P=0.08; [T]P=0.09; CES-D=Center for Epidemiologic Studies Depression Scale; HVLT-R=Hopkins Verbal Learning Test Revised; MNC=mononuclear cells; PSS-10=Perceived Stress Scale; PCL-C=PTSD Checklist Civilian Version; SDMT=Symbol Digit Modalities Test

Supplemental Table 5. Associations (Spearman's Rho) between percentage of mononuclear cells (MNC) TLR2+ (total monocytes), MNC monocyte CD14+CD16- (classical monocytes) , % subset CD14+/CD16+ (intermediate monocytes) , and neuropsychiatric function in women with HIV living in Baltimore, MD (n=25).

	**% MNC**		**% subset**
NP performance	TLR2+	CD14+CD16-	CD14+/CD16+
Global NP function	**0.67****	**0.76*****	**-0.59****
HVLT-R			
Total learning	**0.61****	**0.60****	**-0.70*****
Delayed free recall	**0.72*****	**0.74*****	**-0.77*****
Recognition	**0.59****	**0.63****	**-0.60****
Trail Making Test			
Part A	0.36	**0.52***	**-0.46***
Part B	**0.53***	**0.63****	-0.42†
SDMT	**0.61****	**0.73*****	**-0.53***
Stroop Test			
Trial 2	0.13	0.20	-0.01
Trial 3	**0.55***	**0.63****	-0.28
Grooved Pegboard			
Dominant	0.23	0.31	-0.39‖
Non-dominant hand	0.24	0.33	-0.25
Fluency			
Letter	0.43†	**0.47***	-0.26
Animal	**0.54***	**0.63****	**-0.65****
LNS			
Attention	**0.47***	0.32	-0.09
Working Memory	**0.76*****	**0.77*****	**-0.54***
Mental health			
CES-D	-0.19	-0.17	0.04
PSS-10	-0.15	-0.22	-0.00
PCL-C	-0.08	-0.13	0.22

*P<0.05; **P<0.01; †P=0.06; ‖P=0.08; TP=0.09; CES-D=Center for Epidemiologic Studies Depression Scale; HVLT-R=Hopkins Verbal Learning Test Revised; MNC=mononuclear cells; PSS-10=Perceived Stress Scale; PCL-C=PTSD Checklist Civilian Version; SDMT=Symbol Digit Modalities Test
